# Molecular Imaging of Apoptosis: The Case of Caspase-3 Radiotracers

**DOI:** 10.3390/ijms22083948

**Published:** 2021-04-11

**Authors:** Lucas Beroske, Tim Van den Wyngaert, Sigrid Stroobants, Pieter Van der Veken, Filipe Elvas

**Affiliations:** 1 Molecular Imaging Center Antwerp, University of Antwerp, 2610 Wilrijk, Belgium; lucas.beroske@uantwerpen.be (L.B.); tim.vandenwyngaert@uza.be (T.V.d.W.); sigrid.stroobants@uza.be (S.S.); 2 Department of Nuclear Medicine, Antwerp University Hospital, 2650 Edegem, Belgium; 3 Laboratory of Medicinal Chemistry, University of Antwerp, 2610 Wilrijk, Belgium; pieter.vanderveken@uantwerpen.be

**Keywords:** caspase-3, radiotracer, activity-based probe, substrate-based probe, positron emission tomography, single-photon emission computed tomography

## Abstract

The molecular imaging of apoptosis remains an important method for the diagnosis and monitoring of the progression of certain diseases and the evaluation of the efficacy of anticancer apoptosis-inducing therapies. Among the multiple biomarkers involved in apoptosis, activated caspase-3 is an attractive target, as it is the most abundant of the executioner caspases. Nuclear imaging is a good candidate, as it combines a high depth of tissue penetration and high sensitivity, features necessary to detect small changes in levels of apoptosis. However, designing a caspase-3 radiotracer comes with challenges, such as selectivity, cell permeability and transient caspase-3 activation. In this review, we discuss the different caspase-3 radiotracers for the imaging of apoptosis together with the challenges of the translation of various apoptosis-imaging strategies in clinical trials.

## 1. Introduction

**Figure 1:**
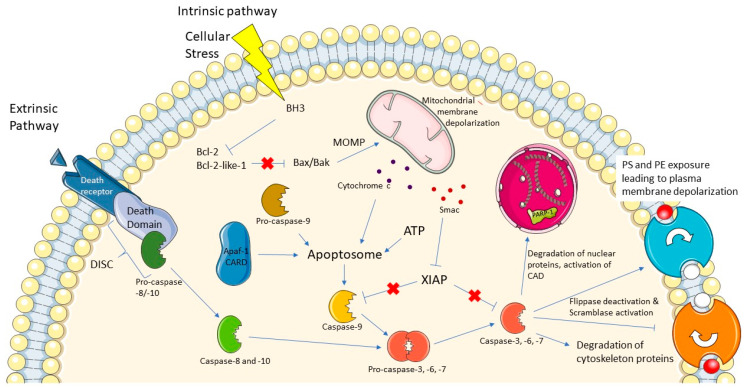
Mechanisms of apoptosis. In the extrinsic pathway, death receptors are activated, leading to the activation of the initiator caspase-8 and -10. In the intrinsic pathway, cellular stress leads to the activation of Bcl-2 homology 3 (BH3)-only proteins, which disable the antiapoptotic proteins Bcl-2 and Bcl-2-like-1 that normally inhibit Bax and Bak proteins. This results in the permeabilization of the mitochondrial membrane, causing mitochondrial membrane depolarization and the release of cytochrome c and Smac proteins. Cytochrome c induces the formation of the apoptosome, followed by the activation of the initiator caspase-9. In both pathways, initiator caspases activate effector caspases, starting the degradation of intracellular material and the death of the cell.

Apoptosis is a programmed form of cell death that plays a vital role in the eradication of aberrant cells that threaten development, homeostasis, and overall survival. This immunologically silent form of cell death can be triggered by the activation of cell surface receptors (e.g., tumor necrosis factor (TNF) receptor superfamily), by release of cytochrome c or DNA damage. Regardless of how apoptosis is initiated, a family of cysteine aspartate-directed proteases, known as caspases, is systematically activated from inactive zymogen-like states, and upon maturation, targets a host of cellular proteins for hydrolysis (Figure 1). The induction of apoptosis leads to both protein substrate degradation and activation, and ultimately cell death [1,2,3,4,5]. Caspases are heavily regulated proteins due to the nature of their activity, as inappropriate activation can have devastating effects. Apoptotic caspases are generally divided into “initiators” and “executioners”. Apoptosis can be activated via two distinct pathways: the extrinsic and intrinsic pathways. The former is triggered via the activation of death receptors, starting a cascade of events that activates the initiator caspase-8. The intrinsic pathway can be induced when the cell is under stress, which leads to the depolarization of the mitochondrial membrane and the subsequent release of pro-apoptotic molecules at the origin of initiator caspase-9 activation (Figure 1). These activated initiator caspases initiate the executioner caspases-3, -6 and -7, with caspases-3 and -7 having redundant roles, and caspase-6 is not essential for apoptosis [1,6].

The dysregulation of apoptosis can lead to various conditions. For example, neurodegenerative diseases, such as Alzheimer’s disease or Parkinson’s disease, are characterized by the loss of neurons, often via excessive apoptosis [7]. Additionally, apoptosis is among the main drivers of diseases such as ischemic heart disease [8]. On the contrary, among the hallmarks of cancer is the ability of cancerous cells to evade apoptosis [9]. For this reason, a common treatment strategy is the induction of apoptosis in tumors, via the activation of the caspase cascade [10,11].

The availability of clinical tools to non-invasively measure apoptosis would represent an important asset in the development of personalized medicine, enabling early disease diagnosis, management, and treatment-response evaluation. Specifically, imaging cell death can have a tremendous impact in the clinic and patients, in particular, in detecting degenerative disorders and predicting treatment failure, such as for certain pro-apoptotic anticancer drugs. Current methods of monitoring therapy response rely on the monitoring of morphological changes using anatomical imaging techniques, such as computed tomography (CT) or magnetic resonance imaging (MRI). The gold standard for the evaluation of tumor response to therapy is the Response Evaluation Criteria in Solid Tumors (RECIST) or immune-related response criteria (irRC) for immunotherapy. However, it can take weeks to months before morphological changes become apparent. Early treatment-response evaluation would save precious time and potential treatment-related toxicity, as well as healthcare resources. Furthermore, it would limit the need for invasive biopsies that may be associated with pain and stress for patients.

While a number of functional imaging techniques exist, positron emission tomography (PET) and single-photon emission computed tomography (SPECT) can be used to detect the spatial and temporal distribution of radioactive probes in vivo accumulating at a target site. PET and SPECT have an extremely high sensitivity, enabling the quantification of small changes in tracer uptake, with multiple radioisotopes with excellent imaging characteristics available for routine clinical use.

For the imaging of apoptosis, caspase-3 represents an attractive target, because it is a specific marker for this mode of cell death. Multiple caspase-3 targeting PET probes have been reported, based on caspase-3 substrates and inhibitors, both small molecules and peptide-derived probes. Typically, these probes consist of a recognition sequence or moiety offering target specificity and selectivity, and a tag, such as a radionuclide, allowing external detection and visualization. However, despite numerous and promising developments, there are limited clinical applications of imaging of apoptosis. In this review, we describe the existing caspase-3 radiotracers, their characteristics and in vitro and in vivo evaluation.

## 2. Challenges of Caspase-3 Probes

Although caspase-3 can be seen as a rather specific marker of apoptosis, it is important to note that apoptosis can be affected by executioner caspases-6 and -7, without the involvement of caspase-3 [12]. Moreover, another regulated mode of cell death called pyroptosis can also be mediated via caspase-3 activation, which was shown to be induced by certain chemotherapy drugs, such as cisplatin [13,14]. Caspase-3 and -7 are highly homologous and share 54% structural identity and 77% active site identity, explaining in part why caspase-3 probes also often target caspase-7 [15]. Both caspase-3 and -7 preferentially recognize and cleave the DxxD motif, a tetrapeptide with two aspartic acid residues, a motif at the basis of many inhibitors and substrates [15,16,17,18]. Each caspase preferentially cleaves a certain substrate. For example, caspase-6 preferentially recognizes the tetrapeptide VExD, while caspase-8 recognizes the LExD motif [18]. As such, achieving the selectivity of caspase-3-targeted probes has proven to be a common hurdle in their development, as other cysteine proteases have similar substrate specificity. Many probes designed to bind caspases will also often target other cysteine proteases, such as cathepsins or legumain, involved in many other processes, including, but not limited to, the recycling of proteins in lysosomes, or the regulation of the immune system [19,20,21]. Efforts to improve the specificity of caspase-3 probes have included extensive structure–activity relationship studies and the introduction of unnatural amino acids into peptide recognition sequences. Moreover, a useful radiotracer tracer must readily cross the plasma membranes to specifically accumulate in cells with high caspase-3 activity, as the target is located in the cytoplasm.

Another challenge that comes with imaging apoptosis is the dynamic nature of the process, which means that the target is only transiently expressed without accumulation, requiring the detection of subtle changes in tracer uptake. Caspase-3 activity peaks 2 to 4 h after the induction of the apoptotic process [22]. Apoptotic cells release “eat-me” signals that promote phagocytosis. Additionally, apoptotic cells that remain undigested then undergo secondary necrosis 12 to 24 h later, when caspase-3 activation is low. This requires the detection of small changes in tracer uptake.

Lastly, the development of radiotracers comes with its own set of challenges. The choice of radionuclide is an important aspect of radiotracer design, which should match the biological half-life of the probe [23]. PET radionuclides generally used in the clinic have short half-lives, the most common being ^11^C (t_1/2_ = 20.4 min), ^18^F (t_1/2_ = 109.7 min) or ^68^Ga (t_1/2_ = 67.6 min), which adds a layer of complexity.

## 3. Small Molecule Probes

### 3.1. Isatin Sulfonamide Family

**Figure 2:**
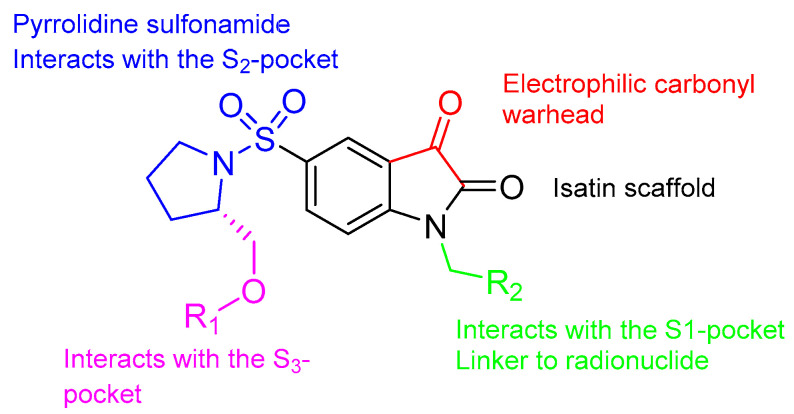
The isatin sulfonamide scaffold. Reprinted with permission from ref. [26]. Copyright 2020 MDPI.

**Figure 3:**
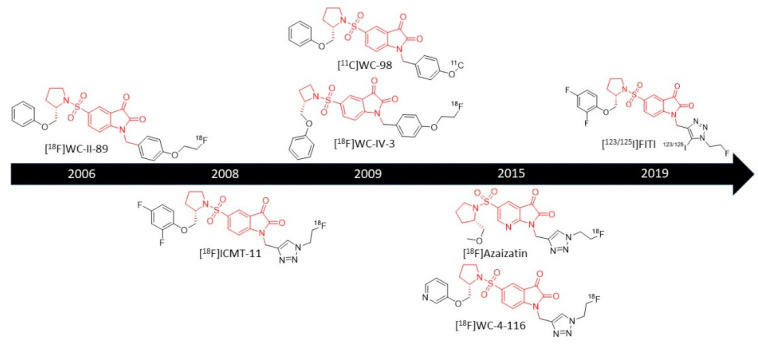
Timeline of the development of the main isatin sulfonamide radiotracers. The core isatin sulfonamide structure is represented in red.

Isatin sulfonamide radiotracers represent an important class of non-peptidic caspase-3/7 activity-based probes (ABPs) (Figure 2). These are small molecules that form a reversible covalent bond in the active site of caspase-3 and -7 with high affinity by nucleophilic attack of the caspase cysteine at the 3-carbonyl group of the isatin moiety, which corresponds to the warhead of the molecule [24,25]. Their nanomolar affinity to caspase-3/7, with IC_50_ values ranging from 0.5 to 80 nM for caspase-3 (Table 1), and their low molecular weight made them good probe candidates to image caspase-3/7 activity [24,26]. They are, however, often prone to metabolic degradation. Several isatin sulfonamide compounds were developed for easy radiolabeling, with either ^11^C, ^18^F or ^123/125^I, often linked to the nitrogen of the isatin scaffold (Figure 3). Radiolabeling was performed either by nucleophilic substitution with direct introduction of fluorine-18, or via a two-step procedure using copper(I)-catalyzed azide-alkyne cycloaddition (CuAAC), between a terminal alkyne on the precursor molecule and 2-[^18^F]fluoroethylazide ([^18^F]FEA). Direct labeling required the protection of the warhead group to avoid any side reactions. In the case of ^11^C labeling, the radiotracer was obtained by O-methylation of a precursor using [^11^C]CH_3_I. Generally, the isatin moiety is kept unmodified, and modifications are allowed at two different positions without significant loss of affinity (R_1_ and R_2_ in Figure 2). Substituents at the isatin nitrogen interact with the S1-pocket, and the introduction of hydrophobic groups at this position was found to improve the affinity to caspase-3 [27]. Moreover, the radionuclide linker was generally attached to the isatin nitrogen. On the other hand, the pyrrolidine was also generally conserved across isatin sulfonamide tracers. It interacts with the S2-pocket of the enzyme via hydrophobic interactions, important for selectivity [25]. Finally, substituents on the pyrrolidine ring interact with the S3-pocket of the enzyme via pi-cation interactions for improved affinity [24,27]. A detailed description of all the modifications performed in the isatin sulfonamide scaffold have been reviewed elsewhere [25].

#### 3.1.1. ^18^F-Labeled Isatin Sulfonamides

##### [^18^F]WC-II-89

[^18^F]WC-II-89 was the first radiolabeled isatin sulfonamide [28]. The radiotracer was obtained via direct labeling by nucleophilic substitution of the mesylate group with fluorine-18. It is highly selective towards caspase-3/7 (IC_50_ values of 9.7 and 23.5 nM, respectively) compared to other caspases (Table 1) [29].

In a hepatic injury rat model, the tracer was shown to accumulate in the liver of cycloheximide-treated rats in a higher proportion to the control, with 0.733%ID/g tissue of tracer uptake in the liver 1 h post-injection (pi), almost a 2-fold increase over untreated rats. Its high lipophilicity (logP = 4.19), however, resulted in the excretion of the tracer via the hepatobiliary route. This probe paved the way for a new class of caspase-3 radiotracers, with improvements made to improve the metabolic stability, the biodistribution profile and the tracer uptake in apoptotic cells.

##### [^18^F]ICMT-11

[^18^F]ICMT-11 was the most promising radiotracer of the isatin sulfonamide class of caspase-3/7 radiotracers. The radiolabeling was first performed via CuAAC with [^18^F]FEA.

The radiotracer showed high affinity and selectivity towards caspases-3 and -7 compared to caspases-1/6/8 (Table 1) [25,30,31].

[^18^F]ICMT-11 was evaluated in many preclinical tumor models, using various cell lines and treatments [26]. These preclinical studies had varying results but generally showed a 1.5- to 2.5-fold higher uptake of the radiotracer in treated tumors compared to controls. It was also compared to the clinical gold standard 2-deoxy-2-[^18^F]fluoro-d-glucose ([^18^F]FDG) in a murine cancer model, where a significant increase in tracer uptake was observed in the tumors of treated mice, while there was a decrease in the uptake of [^18^F]FDG after treatment [30]. [^18^F]ICMT-11 was also shown to be more stable than previous isatin sulfonamides probes, most likely due to the introduction of fluorine atoms on the aromatic ring in the S3-pocket. The logP value of 1.61 is considerably less lipophilic than previously developed isatin sulfonamide caspase-3 tracers, making it more suitable for imaging. Still, in vivo biodistribution showed the excretion of the radiotracer via the hepatobiliary and renal routes [32].

The promising preclinical results led to efforts for the evaluation of [^18^F]ICMT-11 in clinical trials. To achieve radiolabeling under Good Manufacturing Practice (GMP), an alternative strategy had to be used, as the original conditions resulted in a relatively poor specific activity and the presence of a stable impurity [33]. The new strategy consisted of a direct radiolabeling of a tosylated precursor followed by acid treatment to deprotect the dicarbonyl moiety.

**Figure 4:**
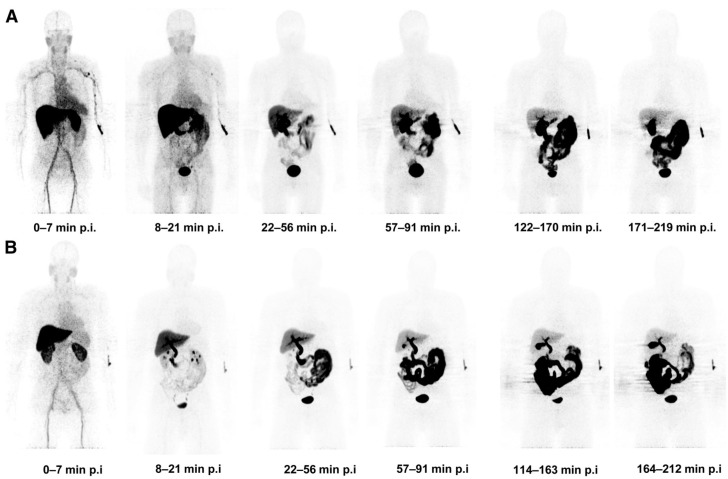
Biodistribution profile of series of [^18^F]ICMT-11. The images correspond to whole-body maximum-intensity projection of representative subjects showing biodistribution of ^18^F activity after tracer injection up to 219 min after injection of [^18^F]ICMT-11. (**A**) A subject who had a meal 2–3 h before tracer injection; (**B**) a subject who had a meal just before tracer injection (due to delays in tracer production). Reprinted with permission from ref. [34]. Copyright 2013 SNMMI.

First, biodistribution and radiation dosimetry studies were performed in 8 healthy volunteers, demonstrating a safety profile similar to other ^18^F PET radiotracers and deemed suitable for imaging in patients, with a mean effective dose of 0.025 ± 0.004 mSv/MBq (0.019 mSv/MBq for [^18^F]FDG) [34,35]. The gallbladder, the small intestine, the upper large intestinal wall, the urinary bladder wall and the liver were the organs that received the highest absorbed dose [34]. However, this tracer showed a suboptimal biodistribution profile, with mixed renal and hepatobiliary excretion, including high uptake in the liver and the intestine, ultimately leading to a high background signal in the abdomen (Figure 4) [34,36].

**Figure 5:**
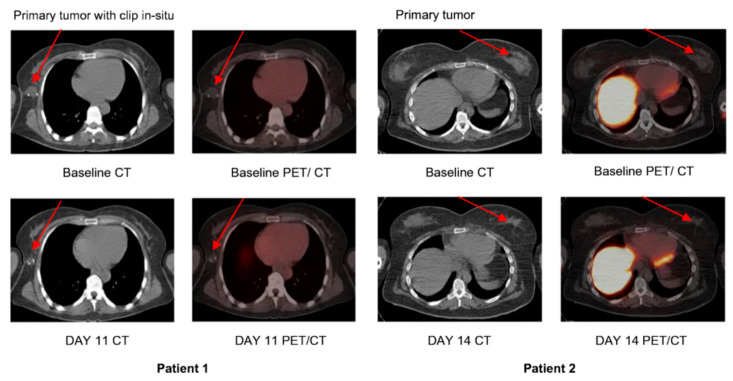
[^18^F]ICMT-11 uptake in primary breast tumors. Axial CT and fused [^18^F]ICMT-11 PET/CT images of primary breast tumors in two patients, 1 and 2, at baseline (pre-) and post-chemotherapy. Low-level uptake is noted. Reprinted with permission from ref. [35]. Copyright 2018 Springer Nature.

Nevertheless, the radiotracer was evaluated in a small cohort of breast and lung cancer patients, before and after first-line chemotherapy. Patients were scanned at baseline as well as 24 h and 7 days after the first cycle of chemotherapy. Overall, low tumor uptake was observed in these patients, which was attributed to a lack of apoptosis induction by the treatment and a heterogeneous response to therapy within tumors (Figure 5) [35]. Moreover, most breast cancer patients had necrotic tumors, explaining the lower levels of tracer uptake. The low tracer uptake in tumors required a PET-based voxel intensity sorting approach to analyze apoptosis levels. This approach showed a higher regional tumor uptake of the radiotracer in patients who responded to the therapy. It is mentioned that this tracer uptake is not an exclusive marker of response, as reviewed by Garcia-Arguello et al., including an extensive analysis of the preclinical and clinical studies of [^18^F]ICMT-11 [26].

##### [^11^C]WC-98

[^11^C]WC-98 is the only ^11^C-labeled isatin sulfonamide probe that was evaluated in vivo. It was obtained via direct labeling by methylation of the phenol, followed by deprotection of the aldehyde in the presence of HCl.

It showed high selectivity towards caspase-3 and -7 with IC_50_ values of 14.5 and 21.8 nM, respectively, while IC_50_ values of over 10 µM for caspase-6, over 20 µM for caspase-1 and over 50 µM for caspase-8 were observed [29]. Additionally, this radiotracer was highly lipophilic with a logP of 3.97.

[^11^C]WC-98 was evaluated in vivo in a comparative study with [^18^F]WC-IV-3 by Chen et al. in a cycloheximide-induced liver apoptosis rat model. An increase in tracer uptake in the liver of Fas antibody-treated mice was observed, albeit to a lower extent than with [^18^F]WC-IV-3, with 1.17%ID/g tissue uptake in the liver of treated rats 30 min p.i., corresponding to a less than 2-fold increase compared to the control. An important drawback of this tracer is the short half-life of ^11^C of 20.4 min, which makes it difficult to detect caspase activation as the background signal would be too high.

##### [^18^F]WC-IV-3

Similar to previous isatin radiotracers, [^18^F]WC-IV-3 was obtained via direct radiolabeling of the mesylate precursor followed by deprotection of the dicarbonyl moiety. To determine the affinity of the inhibitor, the IC_50_ was measured, showing 8.6 and 26.1 nM against caspase-3 and -7, respectively. The IC_50_ for caspase-6 is close to 5 µM and over 20 µM for caspase-1 and -8 [29]. Its logP of 3.65 is indicative of its high lipophilicity.

[^18^F]WC-IV-3 was evaluated in a rat model of liver apoptosis induced by cycloheximide. Chen et al. observed a tracer uptake of 2.75%ID/g tissue in the liver of treated rats 30 min p.i., a 3.5-fold increase compared to the control group. Tracer uptake in the liver correlated with caspase-3 activity in the organ. The tracer was primarily excreted via the hepatobiliary pathway [29]. The stability of the tracer was not reported.

##### [^18^F]WC-4-116

More recently, Chen et al. developed [^18^F]WC-4-116. In vitro characterization showed IC_50_ values of 4.5 and 3.8 nM for caspases-3 and -7, respectively [37]. The tracer showed significant uptake in EL4 murine lymphoma cells treated with etoposide compared to the control, with uptake correlating with caspase-3 activity. Specificity was demonstrated by caspase inhibition with the pan-caspase inhibitor Q-VD-Oph, which resulted in a significant decrease in tracer uptake in etoposide-treated cells.

Biodistribution studies and in vivo metabolic stability studies of the probe showed poor metabolic stability with less than 25% of intact tracer 30 min post-injection and a high background in the abdomen, which can be explained by its lipophilicity (cLogP 1.95) [37,38]. The tracer was also evaluated in a colo205 tumor model treated with the death receptor 5 (DR5)-targeted antibody M413, showing a 1.5-fold higher tracer uptake in tumors with high caspase-3 activity compared to tumors with low caspase-3 activity, with an absolute value of 1.2%ID/cc [37]. Finally, [^18^F]WC-4-116 was evaluated in a myocardial ischemia-reperfusion injury rat model, where it was able to accumulate in myocardial regions with increased levels of apoptosis [39]. Overall, this tracer behaved in a similar way to [^18^F]ICMT-11, with a comparable uptake in treated tumors, and similar metabolic stability [37].

##### [^18^F]Azaisatin

Waldmann et al. developed [^18^F]Azaisatin with the aim to increase the hydrophilicity of the isatin compound and avoid excretion via the hepatobiliary route, with a calculated logD of −1.88 [40]. This was achieved by the use of a 7-azaisatin core and removal of the phenyl ring of other isatin sulfonamide probes at the R1 position. The radiotracer showed a slightly lower potency for caspases-3/7 with IC_50_ values of 21 and 97 nM towards caspase-3 and -7, respectively but increased renal excretion up to 32% compared to 4% for [^18^F]CbR, which was used as a side by side control isatin tracer. Still, the hepatobiliary clearance remained high with 68% excreted via this route, resulting in high background signal in the abdomen. This probe had not yet been evaluated using an in vivo injury/disease model.

##### [^123/125^I]FITI

FITI corresponds to ICMT-11 with an additional iodine on the triazole ring. It was hypothesized that this modification would improve the affinity of the probe towards caspase-3 via the S1 pocket. The K_i_ of FITI against caspase-3 was determined to be 6.1 nM, and the IC_50_ against caspase-8 was over 50 µM, while the corresponding values for ICMT-11 were determined to be 12.4 nM and over 5 µM, respectively. The logD value of 1.6 was the same as ICMT-11. The tracer was obtained by a 3-component CuAAC, which consisted of the reaction of the alkyne precursor, FEA and [^125^I]iodide in the presence of bathophenanthrolinedisulfonic acid (BPDS) [41].

Biodistribution studies showed a high uptake of [^125^I]FITI in the small intestine, the stomach and the liver, indicating a hepatobiliary excretion. The authors hypothesized that the high intestinal tracer uptake could partially be explained by high caspase-3 expression in this region, which could be due to the naturally high turnover of cells on the intestine [41]. [^123^I]FITI was evaluated in a tumor xenograft mouse model treated with etoposide 24 h prior to imaging. The absolute tumor uptake was too low to be visualized in vivo using SPECT imaging, but a significant tracer uptake was detected ex vivo in the tumors of treated mice, with 1.5%ID/g compared to 0.4%ID/g in the control group, 60 min p.i. These results highlight the advantage of PET imaging and the requirements of a highly sensitive imaging technique to detect small changes in caspase-3 activity. Moreover, the probe showed low in vivo metabolic stability, with less than 50% intact tracer 15 min post-injection. The polar metabolite was not characterized. Deiodination was discarded due to the low thyroid uptake. The authors point to a similar polar metabolite for [^18^F]ICMT-11. Taken together, these results suggest that this tracer is not suitable for the detection of caspase-3 in vivo.

Overall, despite promising results and good affinity in vitro, isatin sulfonamide compounds are hampered by several pitfalls: a suboptimal biodistribution profile combined with a low absolute tumor uptake limits their routine clinical application for in vivo imaging of apoptosis [42]. The high tracer accumulation in the abdomen observed in preclinical trials turned out to be important in clinical trials as well. A more hydrophilic probe is necessary to limit its excretion via the hepatobiliary route, especially with such short imaging windows. To tackle the low tumor uptake, Chen et al. also designed polyethylene glycol (PEG)-ylated isatin sulfonamide tracers, which improved cell penetration but also increased the non-specific retention of the tracer, increasing the background signal [37]. Additionally, isatin sulfonamides are generally prone to metabolic degradation, although [^18^F]ICMT-11 is more stable. Finally, there are concerns about the non-specific reactivity of the dicarbonyl moiety towards other cysteine proteases, such as cathepsins or other nucleophiles, an aspect that requires further investigation [43].

### 3.2. Other Small-Molecule Radiotracers

#### [^18^F]pyrimidoindolone

[^18^F]pyrimidoindolone represents a new generation of caspase-3/7 probes. Similar to the isatin sulfonamide probes, it relies on an electrophilic carbonyl to bind caspase-3/7. However, the reactivity of the warhead was tuned down to limit non-specific reactions by using a pyrimidoindolone instead of the dicarbonyl of isatin compounds [44]. Additionally, a spirocyclopentyl ring was introduced at the P1 residue to improve aqueous stability. The reported IC_50_ of this inhibitor was 100.4 nM against caspase-3, two orders of magnitude lower affinity than ICMT-11. To evaluate the selectivity of the pyrimidoindolone, IC_50_ was only measured against caspase-8, with a value over 5 µM. The affinity of the inhibitor was not determined for other caspases or even other types of proteases.

The cell uptake of [^18^F]pyrimidoindolone was evaluated by incubating the tracer with 38C13 lymphoma cells treated with 4-hydroperoxycyclophosphamide to induce apoptosis. A 1.7-fold increase in tracer uptake in treated cells was observed compared to the control [44].

Biodistribution studies showed a high tracer uptake in the kidneys, urine, liver and intestine, indicating a mixed hepatobiliary and renal excretion of the probe, similar to that of [^18^F]ICMT-11. Additionally, plasma stability studies revealed that the new probe was more prone to metabolic degradation, with 14% intact tracer remaining in plasma after 60 min, compared to 65% of intact [^18^F]ICMT-11 [44]. Overall, the lower affinity of the probe was thought to be partially counteracted by its ability to bind both subunits of caspase-3/7, which is not the case for the isatin sulfonamide tracers, thus doubling the potential binding sites.

## 4. Peptide-Based Caspase-3 Probes

Another class of caspase-3 targeting PET probes includes peptide derivatives. These are based on either caspase-3 substrates (substrate-based probes or SBPs) or inhibitors (ABPs). SBPs are probes that can be cleaved by the target enzyme. Upon cleavage of the substrate, the portion containing the tag is trapped inside the cell. On the contrary, ABPs contain an electrophilic warhead for covalent binding to the target. The added steric hindrance and electronics of an electrophilic warhead moiety of ABPs can also confer additional selectivity to the probes towards the desired target by interacting with the prime side of the enzyme when combined with a non-prime side directed peptide, as described by Vickers et al. [17,45]. Common electrophilic warheads used in caspase-3 ABPs are acyloxymethyl ketone (AOMK) and fluoromethyl ketone (FMK) groups, as they are specific for cysteine proteases [19].

It has been hypothesized that the ABP approach would not yield sufficient target accumulation and that the resulting signal could be too low to distinguish from the background signal [46]. Substrate-based probes were thought to be a better alternative, potentially leading to the accumulation of radioactivity in cells with higher caspase-3 activity, resulting in signal amplification. In practice, however, SBPs do not necessarily lead to an increased signal, as described by Blum et al., due to diffusion of the cleavage fragment away from the protease [19,47]. A higher signal was observed in a comparative study of ABPs and SBPs in a mouse tumor model with an ABP, which was attributed to better kinetics and better retention of the probe in the tumor. Therefore, for successful visualization of an SBP, the cleaved labeled substrate must be trapped inside the cell. Clever designs include the formation of nanoaggregates or the use of the change in pH between the extracellular space and organelles within the cell [48,49]. The first DEVD-based radiotracers were developed by Bauer et al. They fitted a series of peptides containing the DEVDG sequence with various tat peptides, a type of cell-penetrating peptide (CPP), to improve cell penetration. The probes were radiolabeled with ^131^I and chloramine-T by electrophilic radioiodination of a tyrosine in each sequence [50]. They showed accumulation of their probes in apoptotic cells in vitro and served as an interesting proof of concept for further research.

### 4.1. Substrate-Based Probes

#### 4.1.1. [^18^F]CP-18

[^18^F]CP-18 is a substrate-based probe targeted to caspase-3/7. The structure of this tracer consists of the pentapeptide DEVDA for recognition by caspase-3/7 fitted with a galactose moiety on the N terminus for fast renal clearance and a PEG chain on the C terminus to facilitate cell uptake. Radiolabeling was obtained by CuAAC between 5-[^18^F]fluoro-1-pentyne and an azide linked to the sugar moiety of the precursor. Upon recognition by caspase-3/7, the PEG chain is cleaved from the rest of the molecule, resulting in a less lipophilic molecule and subsequent trapping and accumulation of the radiolabeled moiety inside the cell [51,52,53]. In vitro evaluation of the probe showed significant uptake of the tracer in treated cells compared to controls across different cancer cell lines: colorectal, lung carcinoma and glioblastoma cells treated with 5-fluorouracil or irinotecan [52,54].

In vivo, [^18^F]CP-18 was evaluated in a dexamethasone-induced thymic apoptosis mouse model, a model of chronic anthracycline-induced cardiotoxicity as well as different xenograft tumor models [51,52,54,55]. In the first two models, a significant tracer uptake was observed in the groups where apoptosis was induced compared to the control groups [51,55]. More recently, [^18^F]CP-18 was evaluated in a colorectal cancer xenograft model, where colo205 cell-bearing mice were treated with 5-fluorouracil (5-FU), irinotecan or their combination [52]. This study showed the significant accumulation of the tracer in tumors of mice with the combination therapy, albeit with a low absolute uptake (0.60 ± 0.32%ID/cm^3^). This was partially due to low levels of apoptosis induced by the treatment. Moreover, doubt was cast on its specificity towards caspase-3/7: Rapic et al. showed that 5-FU induced apoptosis via a caspase-9-dependant pathway, and without caspase-3 activation, suggesting increased tracer uptake in tumors due to the recognition of other caspases [52].

**Figure 6:**
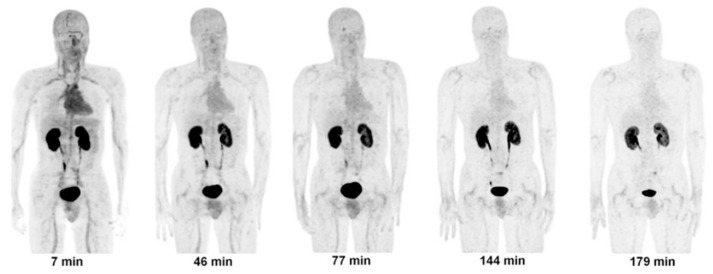
Decay-corrected anterior maximum-intensity projections of PET at 7, 46, 77, 144 and 179 min (from left to right) after injection of [^18^F]CP-18 in male volunteer. There was rapid clearance of activity in all organs. Reprinted with permission from ref. [53]. Copyright 2013 SNMMI.

[^18^F]CP-18 was also evaluated in humans. First, Doss et al. evaluated the biodistribution and dosimetry profiles of the radiotracer in healthy volunteers, which showed rapid clearance primarily via the kidneys (Figure 6). They showed that the tracer was safe, with most of the radiation absorbed by the urinary bladder wall, the kidneys and the uterus. The effective doses (µSv/MBq) were 38 ± 3.9 for 4.8 h and 15 ± 1.9 for 1 h void intervals, which is comparable to the typical injection dose of [^18^F]FDG (20 µSv/MBq for an injection of 455MBq) [56]. In vivo stability showed the formation of metabolites due to the cleavage of the DEVD sequence, with 37 and 21% of the intact tracer after 35 and 135 min, respectively. The parent tracer and metabolites were all primarily excreted via the kidneys relatively quickly, with 77% of the activity excreted in 2.8 h [53]. The evaluation of [^18^F]CP-18 in a phase II clinical trial in patients with ovarian, fallopian tube or peritoneal cancer treated with Birinapant was registered in 2013 (NCT01766622), but was subsequently withdrawn for unknown reasons.

#### 4.1.2. [^18^F]TBD

[^18^F]TBD is a caspase-3 selective substrate obtained by CuAAC between [^18^F]FEA and an alkyne precursor, and purified by solid-phase extraction, enabling a fast purification method [57]. The probe was based on the pan-caspase covalent inhibitor M808, containing two amino acids, aspartic acid and alanine, and the warhead FMK, which was optimized by combinatorial studies [58]. Different analogs were tested against caspase-3 to evaluate substrate activity and selectivity, leading to their final compound, the dipeptide containing aspartic acid and the unnatural amino acid threonine O-benzyl ester. The fluorescent version of this substrate was shown to be selective towards caspase-3 compared to a panel of caspases. It was hypothesized that the lower molecular weight of this SBP compared to DEVD-based probes would improve its cell permeability. The measured logP of [^18^F]TBD of 0.75 makes it a rather lipophilic compound, albeit to a lower extent than previously developed isatin sulfonamides. The optimized dipeptide was able to accumulate in apoptotic liver cells treated with cisplatin, correlating with the concentration of the drug in a dose-dependent manner.

For biodistribution studies, only one time-point was used. After 35 min post-injection, there were low levels of tracer in the small and large intestine with less than 2%ID/g in the treatment group. The tracer was shown to be excreted via mixed renal and hepatobiliary routes. The tracer was also evaluated in vivo in a Jo2-induced hepatic cytotoxicity model. The mice were treated with the pro-apoptotic Jo2 antibody, which targets CD95 and readily accumulates in the liver. The mice were injected 2 h post-treatment and scanned for 30 min. These results show higher accumulation of the tracer in the liver of treated mice (ex vivo accumulation slightly over 2%ID/g) than non-treated mice (ex vivo accumulation around 1.5%ID/g). A control probe was used, which showed less accumulation in the treated mice than in the control. Immunohistochemistry on liver slices also showed high levels of cleaved caspase-3 two hours after treatment, which appeared to be greater than the corresponding tracer accumulation.

#### 4.1.3. [^68^Ga]Ga-TC3-OGDOTA

[^68^Ga]Ga-TC3-OGDOTA is a dual radioactive and fluorescent probe designed to accumulate in apoptotic cells [59]. It was designed to enable the crossing of the blood–brain barrier to visualize apoptosis in the brain for the diagnosis of neurological disorders, such as Alzheimer’s disease or stroke. This tracer contains the DEVD sequence, an HIV1 tat-based cell-penetrating peptide (CPP), a DOTA chelator for ^68^Ga radiolabeling and an Oregon green as fluorescent moiety. The radiotracer was obtained in two steps. First, the fluorophore was coupled to the peptide containing DEVD, the CPP and DOTA by nucleophilic substitution at a cysteine side chain. DOTA and the fluorophore are both connected to a cysteine located on one side of the DEVD recognition sequence, while the CPP is on the other side of the recognition sequence. Then, the precursor was radiolabeled with ^68^Ga.

In vitro, the probe selectively accumulated in neurons treated with the pro-apoptotic compound camptothecin, and prevented when using a caspase-3 inhibitor. Accumulation of the probe was validated by fluorescence microscopy. The caspase-3 tracer also accumulated in cell culture models of stroke and Alzheimer’s disease.

The probe was then investigated in two animal models: a cerebral ischemia model and an Alzheimer’s disease model. In both models, the probe was able to accumulate in areas with increased apoptosis.

This tracer presented some drawbacks: Ostapchenko et al. mention the poor resolution of the images causing difficulty to distinguish the uptake in different brain structures, and the stability of the tracer was not determined. Moreover, the brain could only be harvested for confocal microscopy after decay of the radionuclide, which could have affected the distribution of the probe in the brain [59].

### 4.2. Activity-Based Probes

#### 4.2.1. [^18^F]FB-VAD-FMK

[^18^F]FB-VAD-FMK is a radiolabeled ABP targeting caspases derived from the covalent pan-caspase inhibitor Z-VAD-FMK, consisting of the peptide sequence Val–Ala–Asp, the FMK warhead and the ^18^F tag [60]. The probe was radiolabeled by nucleophilic aromatic substitution of the prosthetic group, followed by the deprotection of the carboxylic acid and the formation of the activated ester, obtaining [^18^F]N-succinimidyl-4-fluorobenzoate ([^18^F]SFB). The prosthetic group was then coupled to VAD-FMK in a 45 min reaction to form the desired radiotracer. This multistep radiosynthesis is time-consuming (with a total preparation of over 100 min), making its routine production difficult. Z-VAD-FMK has shown high affinity throughout the caspase family, with a nanomolar potency against caspases-1/5/8/9, and is commonly used to inhibit apoptosis in vitro [61]. The lack of caspase selectivity of Z-VAD-FMK makes it unlikely that the new probe selectively targets only caspases involved in apoptosis. The radiotracer showed low absolute tumor uptake in different mouse models treated with the Aurora kinase B inhibitor AZD-1152 (0.79%ID/g) or the combination therapy of the phosphoinositide 3-kinase inhibitor BEZ-235 and BRAF inhibitor PLX-4720 (1.54%ID/g). Additionally, the radiotracer showed high background, especially in the liver and kidneys, denoting a mixed hepatobiliary and renal excretion, all of which make it a suboptimal radiotracer for cell death imaging [60].

#### 4.2.2. [^18^F]MICA-302

**Figure 7:**
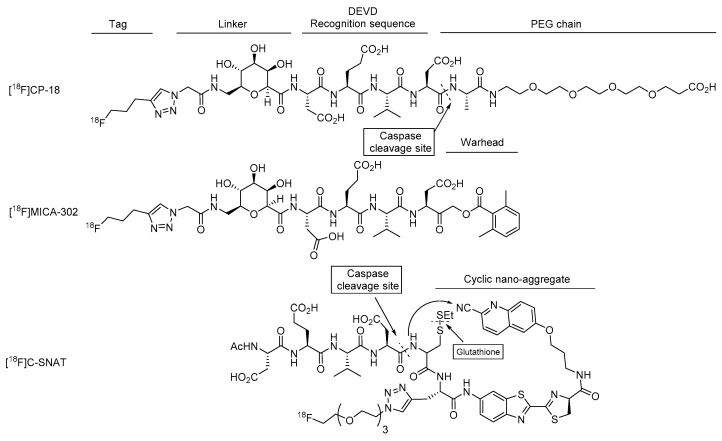
Molecular structure of DEVD-based radiotracers: [^18^F]MICA-302 ABP, [^18^F]CP-18 SBP and [^18^F]C-SNAT SBP.

[^18^F]MICA-302 is a peptide-based ABP (Figure 7). It consists of the DEVD tetrapeptide recognition sequence for caspase-3/7 and the C-terminal electrophilic warhead AOMK at the C terminus and a N terminal [^18^F]fluorinated triazole moiety, obtained by CuAAC between a 5-[^18^F]fluoro-1-pentyne and an azide precursor. Similar to CP18, MICA-302 contains a sugar linker to increase polarity and favor renal excretion of the probe, as shown by the logD of -3.09. Several electrophilic warheads were tested, showing that less reactive and bulkier electrophilic warheads conferred more affinity and selectivity towards caspase-3 compared to other cysteine proteases [16]. The corresponding inhibitor showed high affinity towards caspases-3/7, with reported IC_50_ values of 1.0 and 13.2 nM towards human caspase-3 and -7, respectively.

In vivo evaluation of the probe showed high metabolic stability and good biodistribution with fast renal excretion, fast clearance from the blood and only a limited accumulation in the small intestine with less than 4%ID/g 60 min p.i. due to the high hydrophilicity of the probe with a logD of −3.09. However, similar to previously developed tracers, low absolute tumor uptake was observed in vivo after treatment with conatumumab (1.52%ID/mL) 24 h, which was attributed to the poor cell permeability of the tracer [16]. Additionally, a control probe was used with a similar structure to MICA-302. This probe did not significantly accumulate in tumors of treated mice compared to untreated mice. Therefore, additional chemical modifications might be required to enhance cell penetration when targeting caspase-3 in live animals.

Peptide-based SBPs and ABPs, despite promising results in vivo and satisfactory biodistribution profiles, have low tumor uptake due to low cell permeability or low levels of apoptosis, making it difficult to translate into the clinic. Potential strategies to improve tumor uptake include the use of cell-penetrating peptides, or the tuning of the properties of radiotracers by modifying the linker. To improve upon ABPs, the Wolan group designed a series of selective caspase-3 inhibitors. A first generation was developed with the introduction of unnatural amino acids in the DEVD sequence, obtaining Ac-DW3-KE [45]. This promising inhibitor turned out to have a major limitation: its binding kinetics proved to be too slow for in vivo use. Therefore, a second generation of fast-binding selective caspase-3 inhibitor was designed, termed Ac-ATS010-KE, with the potential to be used as a potent, highly selective, fast-binding radiotracer [17].

### 4.3. Nanoaggregate Probes

#### 4.3.1. [^18^F]C-SNAT

[^18^F]C-SNAT is a DEVD-based smart probe that is activated in the presence of caspase-3/7 and glutathione. The mechanism of activation of this probe consists of the cleavage of the DEVD motif and the reduction in the cysteine disulfide bond, allowing intramolecular cyclization via a condensation reaction among the free thiol, the free amine and the nitrile. This results in the nanoaggregation of the cyclic probes into nanoparticles via hydrophobic interaction, preventing them from exiting the cell (Figure 7). The radiolabeling of the probe was performed in two steps. First, the tosylated azido-triehtylene glycol precursor was radiolabeled with [^18^F]. The resulting prosthetic groups were then purified by semi-preparative HPLC and coupled to the C-SNAT via CuAAC [62]. Overall, the radiosynthesis of [^18^F]C-SNAT takes 3–3.5 h, an aspect that should be improved to be used in the clinic [63].

Incubation of the probe in HeLa cells treated with Doxorubicin resulted in an over two-fold increase in tracer uptake compared to the control, which was partially prevented with the addition of a pan-caspase inhibitor [64]. In experiments performed by Palner et al. and Witney et al., using xenograft mouse models treated with doxorubicin or clinically formulated etoposide, [^18^F]C-SNAT could accumulate in treated tumors, with absolute tumor uptake averaging 1–3%ID/g [49,63]. Witney et al. performed a comparison study of [^18^F]C-SNAT with [^18^F]FDG, and two other apoptosis probes, [^99m^Tc]Annexin V and [^18^F]ML-10. They only saw a significant difference in tracer uptake in treated mice compared to the control with [^99m^Tc]Annexin V and [^18^F]C-SNAT. The probe had a combined hepatobiliary and renal excretion. Additionally, pharmacokinetic studies of the probe show increased accumulation in treated tumors combined with a small dissipation rate [49]. The probe was stable in mouse plasma. However, after 2 h, the majority of the probe had already been reduced. However, this metabolite should also accumulate in apoptotic cells [49].

#### 4.3.2. DEVD-Cys(StBu)-PPG(CBT)-AmBF_3_

Qiu et al. designed a DEVD-based probe inspired by [^18^F]C-SNAT, consisting of the DEVD recognition sequence, a protected cysteine, the radioactive tag [^18^F]AmBF_3_ enabling a more rapid radiolabeling and a 2-cyanobenzothiazole (CBT) group. Upon the reduction in the protected cysteine (by glutathione for example), an intermolecular nucleophilic attack of the cysteine side chain to the electrophilic CBT can occur, resulting in the formation of a dimer prone to forming nanoaggregates via π-π stacking interactions. The large size of the nanoaggregates prevents them for leaving the cell, resulting in an amplified signal in apoptotic cells.

The main difference between the two probes is the radiolabeling method. The radiolabeling and purification could be performed in 25 min with high radiochemical yield and purity, due to a one-step radiolabeling via isotopic exchange radiolabeling in mild conditions and SPE purification, with a specific activity of 1.45 ± 0.4 Ci/µmol. The specific activity is relatively low. This is due to the fact that it is impossible to purify the radioactive tracer from the non-radioactive precursor, as they are identical.

In vitro evaluation of the radiotracer indicated the reduction in and cleavage of the probe in the lysates of HeLa cells, with the formation of a dimer, enabling the formation of nanoaggregates. Furthermore, the probe showed high stability in mouse plasma with more than 95% of intact tracer after 4 h. The tracer also accumulated in HeLa cells treated with doxorubicin, which was prevented when using the pan-caspase inhibitor zVAD-fmk.

The in vivo evaluation of this tracer was promising with high accumulation in the tumor and a mixed hepatobiliary and renal excretion. In a xenograft cancer mouse model, HeLa tumor cell-bearing mice were first treated with an intratumoral injection of doxorubicin. Co-injection of the radiolabeled tracer and the non-radiolabeled precursor led to a higher tumor uptake and better tumor-to-muscle ratio than the tracer alone (T/M ratio of 2.18, 10.52 and 14.81 after 60 min for untreated, tracer alone and tracer and non-radioactive probe, respectively). This phenomenon was thought to be due to the non-radioactive compound promoting the formation of the nanoparticles with the radioactive probe. A follow-up study with intravenous injection of doxorubicin in the same mouse model showed higher tumor uptake of the tracer in treated mice compared to untreated mice (T/M ratio of around 1, 4.24 and 5.62 after 60 min for untreated, tracer alone and tracer and non-radioactive probe, respectively). A similar trend was observed, although uptake was lower with a peak tumor accumulation of 3.22 ± 0.25 and 4.08 ± 0.22 %ID/mL at 15–20 min for the tracer alone and co-injected with the cold precursor, respectively. These findings were explained by lower levels of apoptosis induced by the treatment via intravenous injection compared to intratumoral injection. The authors explain the better results with co-injection of the cold precursor by the better self-assembly of the nanoparticles.

#### 4.3.3. TCO-C-SNAT4

TCO-C-SNAT4 uses a pre-targeting strategy where the substrate can accumulate in cells with active caspase-3/7 and allows time to wash out before the injection of the radiolabeled tag [65]. The tetrazine (Tz) tag then reacts in vivo with the free trans—cyclooctene (TCO) via a biorthogonal inverse—electron demand Diels–Alder reaction (IEDDA).

Chen et al. showed that the cleavage of DEVD and reduction in cysteine disulfide bride lead to internal cyclization of the molecule and subsequent aggregation into nanoparticles, the formation of which was confirmed by dynamic light scattering and transmission electron microscopy. The probe was evaluated using a fluorescent Tz in two cell lines in which apoptosis was induced by cisplatin or staurosporine. The probe accumulated in apoptotic cells, which was prevented when pre-incubating with a covalent caspase-3 inhibitor.

This strategy was evaluated in vivo in a xenograft tumor model. Mice with H460 tumor cells were treated with cisplatin, and injected with TCO-C-SNAT4 24 h later. After 30 min, the Tz radiolabeled with ^64^Cu was injected and the mice immediately scanned. The tracer uptake in the tumors of treated mice (3.5 ± 0.3% ID/cc) was significantly higher than in non-treated mice (1.4 ± 0.1% ID/cc. Biodistribution studies show excretion of the probe primarily via the renal pathway. The radionuclide ^64^Cu with a long half-life of 12.7 h was chosen to investigate the biological half-life of the nanoaggregates. Further research with an optimized timing for imaging and the use of other radionuclides, such as ^18^F, could be of interest. Moreover, the TCO, which requires transconformation to readily react to the Tz, was shown to revert to the cis-formation in vivo, with a half-life of around 4 h. Therefore, the inverted approach where the Tz is coupled to C-SNAT4, and the radiolabeled TCO injected at a later time point, could potentially improve the tracer uptake in vivo.

The different nanoaggregate probes show promising results with significant uptake and retention in treated tumors. Moreover, the quick radiolabeling and ease of purification of the latest generations make this type of probe more suitable for routine production. The pre-targeting strategy could be of great interest to increase the amount of accumulated nanoaggregates in the tumor, especially with imaging at later time points given that the nanoparticles remain in the tumor. Therefore, more research is necessary to evaluate the outcomes of the nanoaggregates at a later stage.

## 5. Conclusions and Further Perspectives

Despite the considerable amount of research, the development of effective caspase-3 radiotracers faces three main hurdles: (1) low absolute tumor uptake in different disease models, (2) high background signal due to suboptimal biodistribution profiles and (3) lack of target selectivity. Overcoming all these hurdles is a difficult task. Effective caspase-3 probes must combine and balance several factors, including high metabolic stability, fast tumor uptake and caspase-3 inhibition, high affinity and selectivity towards caspase-3 and fast renal clearance. Every attempt at solving one of these aspects results in a slightly less optimal feature in another aspect. Importantly, the selectivity of the tracers should be investigated more thoroughly, as many studies limit their targets to caspases. The risk with off-target binding is the increase in background due to the imaging of processes not involved in the apoptotic process.

There are different strategies to tackle cell uptake, including several probes fitted with CPP, such as the first DEVD-based probe [50]. Additionally, pre-targeting strategies are increasingly being investigated, as they allow longer circulation times of the targeting moiety, while the tag can be injected at a later time point, enhancing the signal-to-background ratio. In order to improve the biodistribution profile of caspase-3 probes, more hydrophilic compounds have been developed, such as CP-18 or MICA-302, with the addition of a sugar linker.

There are additional challenges when imaging apoptosis. First, the dynamic nature of apoptosis (and caspase activity) represents an intrinsic limitation for imaging, making the timing of imaging of the utmost importance to obtain a high signal in the region of interest. This is especially challenging in a clinical setting where the heterogeneity of patients and tumors influences the peak apoptosis, and where patients can only be scanned for a limited amount of time. Moreover, short imaging windows lead to greater susceptibility to variability [66]. Another important point is that in an ideal world, the radiotracer would quickly accumulate in the site of interest, whereas the unbound fraction is quickly excreted to obtain a high signal-to-background ratio. However, the gut naturally has a high turnover of cells with higher levels of apoptosis than other tissues. This means that caspase-3 probes will accumulate in the gut, resulting in a high background signal. An observation is that the uptake in the small and large intestines is rarely reported in ex vivo biodistribution studies.

Overall, the apoptotic tumor response to therapy is a complex process. Other approaches for imaging apoptosis have been investigated, targeting multiple apoptosis events, such as the loss of mitochondrial membrane potential, DNA fragmentation and loss of plasma membrane asymmetry [67,68,69]. Nevertheless, due to its specificity, activated caspase-3 remains an attractive target for the imaging of apoptosis for treatment-response evaluation. The evaluation of only two caspase-3/7 radiotracers—the isatin sulfonamide [^18^F]ICMT-11 and the DEVD-based SBP [^18^F]CP-18—in clinical trials shed light on how difficult the task of visualizing apoptosis in humans can be. Generally, low tracer uptake in tumors can be explained by a multitude of reasons. This can be due to poor cell permeability, relatively low levels of apoptosis induced by the treatments or the poor vascularization of the tumors preventing enough tracer reaching the region of interest. The future of caspase-3 imaging will depend on the optimization of the probes to improve delivery to apoptotic cells while keeping the background signal to a minimum.

## Figures and Tables

**Table 1:** Caspase-3 affinity of various isatin sulfonamide inhibitors and peptide-based ABPs, their relative selectivity and log P.

Inhibitor	Caspase-3 Affinity IC_50_ (nM)	Selectivity Index					Log P
		−1	−6	−7	−8	−9	
WC-II-89	9.7	>5000	>361	2.4	>5000	N/A	4.19
ICMT-11	0.5	>10,000	>10,000	5.0	>10,000	N/A	1.61
WC-98	14.5	>1300	>680	1.5	>1300	N/A	3.97
WC-IV-3	8.6	>2300	580	3.0	>2300	N/A	3.65
WC-4-116	4.5	>4400	2989	0.8	>11,000	N/A	0.73 (calculated)
Azaisatin	21	>2380	>2380	4.6	N/A	N/A	−1.32
FITI	Ki 6.1	N/A	N/A	N/A	>8190	N/A	1.6
Pyrimidoindolone	100.4	N/A	N/A	N/A	>49.8	N/A	N/A
FB-VAD-fmk	225.0	N/A	N/A	N/A	N/A	N/A	N/A
MICA-302	1.0	1000	20	13.2	23.6	>1000	−3.09
